# Long-Term Outcomes Associated With Permanent Pacemaker Implantation in Low-Risk Surgical Aortic Valve Replacement

**DOI:** 10.1016/j.jacadv.2024.101110

**Published:** 2024-07-18

**Authors:** Ruixin Lu, Natalie Glaser, Ulrik Sartipy, Michael Dismorr

**Affiliations:** a Department of Molecular Medicine and Surgery, Karolinska Institutet, Stockholm, Sweden; b Department of Cardiology, Stockholm South General Hospital, Stockholm, Sweden; c Department of Cardiothoracic Surgery, Karolinska University Hospital, Stockholm, Sweden

**Keywords:** all-cause mortality, endocarditis, heart failure hospitalization, low surgical risk, permanent pacemaker implantation

## Abstract

**Background:**

Permanent pacemaker implantation is associated with an increased risk of mortality and heart failure after surgical aortic valve replacement (SAVR).

**Objectives:**

The purpose of this study was to analyze long-term prognosis of permanent pacemaker implantation following SAVR on low-risk patients.

**Methods:**

This nationwide, population-based, observational cohort study included all patients who underwent SAVR in Sweden between 2001 and 2018 with low surgical risk, defined as logistic EuroSCORE I <10% or EuroSCORE II <4%. Patients received a permanent pacemaker implantation within 30 days after SAVR. Main outcomes were all-cause mortality, heart failure hospitalization, and endocarditis. Regression standardization addressed confounding.

**Results:**

We included 19,576 patients with low surgical risk. Of these, 732 (3.7%) patients received a permanent pacemaker within 30 days after SAVR. The mean age was 68 years and 33% were women. We found no difference in all-cause mortality between patients who received a pacemaker compared to those who did not (absolute survival difference at 17 years: 0.1% (95% CI: −3.6% to 3.8%). After 17 years, the estimated cumulative incidence of heart failure in patients who received a pacemaker was 28% (95% CI: 24%–33%) vs 20% (95% CI: 19%–22%) in patients who did not (absolute difference 8.2% [95% CI: 3.8%–13%]). We found no difference in endocarditis between the groups.

**Conclusions:**

We found an increased incidence of heart failure in patients with low surgical risk who received a permanent pacemaker after SAVR. Permanent pacemaker implantation was not associated with all-cause mortality or endocarditis. Efforts should be made to avoid the need for permanent pacemaker following SAVR.

Permanent pacemaker implantation is a known complication after both surgical aortic valve replacement (SAVR) and transcatheter aortic valve replacement (TAVR). The prevalence of permanent pacemaker implantation after SAVR ranges between 3% to 5%, and between 9% to 26% after TAVR.^1^, ^2^, ^3^ A previous study by our group showed that postoperative permanent pacemaker implantation after SAVR was associated with long-term mortality and increased rates of heart failure.^1^ The long-term effects of permanent pacemaker implantation after TAVR may not be as severe.^4^ This might be explained by an older patient population with a higher surgical risk undergoing TAVR than SAVR, where other factors such as comorbidity and age may have a more important impact on survival and other long-term clinical outcomes. However, the effect of permanent pacemaker implantation after SAVR in patients with low surgical risk is not known. Permanent pacemaker implantation in patients with low surgical risk may have a greater influence on long-term prognosis due to the low burden of comorbidities, compared to patients with a higher surgical risk. The impact of permanent pacemaker implantation in patients with low surgical risk is of increasing importance, both in terms of clinical decisions for the individual patient but also owing to the growing number of patients with low surgical risk subject to TAVR procedures.^1^^,^^4^^,^^5^ We therefore performed a follow-up study of our previous study^1^ to investigate the prognosis after permanent pacemaker implantation following SAVR in the subset of patients who had low surgical risk.

## Material and methods

### Study design

This was an observational, nationwide, population-based cohort study. This study was approved by the Swedish Ethical Review Authority and the requirement for informed consent was waived (registration number: 2020-04967). Study reporting followed the STROBE (Strengthening the Reporting of Observational studies in Epidemiology) and RECORD (REporting of studies Conducted using Observational Routinely collected health Data) guidelines.^6^^,^^7^

### Study population and exposure

The study included all patients with low surgical risk who underwent primary SAVR in Sweden between January 1, 2001, and December 31, 2018. Low surgical risk was defined as a logistic EuroSCORE I <10%, for patients operated between 2001 and 2011, or EuroSCORE II <4%, for patients operated between 2012 and 2018.^8^ Logistic EuroSCORE I was used for patients operated between 2001 and 2011 and EuroSCORE II for patients operated between 2012 and 2018 because EuroSCORE II was not introduced into Swedish practice until 2012. Exposure was defined as permanent pacemaker implantation within 30 days following SAVR. Patients were excluded if they met any of the following criteria: died within 30 days of AVR, had a pre-existing permanent pacemaker or an implantable cardioverter defibrillator, logistic EuroSCORE I ≥10%, EuroSCORE II ≥4%, previous cardiac surgery, previous TAVR, preoperative endocarditis, concomitant surgery on another valve, emergent surgical treatment (ie surgery within 24 hours from the decision to operate), or the use of deep hypothermia and circulatory arrest. Swedish Classification of Health Interventions (adapted from the NOMESCO Classification of Surgical Procedures) was used to classify the exposure, baseline, and outcome procedures. The study population constitutes a subgroup of our previous study,^1^ including only patients with low surgical risk. All patients in this study were included in our previous study.

### Outcomes

The primary outcome was all-cause mortality, obtained from the Swedish Total Population Register.^9^ Secondary outcomes were the cumulative incidence of heart failure hospitalization and endocarditis, obtained from the National Patient Register.^10^ The corresponding International Classification of Diseases (ICD)-9 and ICD-10 codes were used to ascertain the secondary outcomes and exposure was obtained from the National Patient Register. The ICD codes used are presented in Supplemental Table 1.

### Data sources

The Swedish Cardiac Surgery register, which is a part of the SWEDEHEART (Swedish Web-system for Enhancement and Development of Evidence-based care in Heart disease Evaluated According to Recommended Therapies) registry, was used to identify the study population.^11^ The Swedish Cardiac Surgery register records all patients who have undergone cardiac surgery in Sweden since 1992, and contains preoperative, perioperative, and postoperative data, including survival status through linkage with the Total Population Register. The Swedish Cardiac Surgery register has high reliability and validity.^12^ The Swedish National Patient Register was used to obtain additional baseline characteristics, and outcome data for the hospitalization for heart failure and endocarditis outcomes. The heart failure diagnose has high reliability and high validity in the National Patient Register during external review.^10^^,^^13^ Socioeconomic background characteristics were obtained from the Longitudinal Integrated database for health insurance and Labour market Studies, maintained by Statistics Sweden.^14^ The Swedish Personal Identity Number made it possible to cross-link data at an individual level.^15^

### Statistical methods

Categorical baseline characteristics were presented as frequencies and percentages, continuous variables were presented as mean ± SD. The time-to-event was defined as the number of days from the date of surgery until the date of event, or end of follow-up, whichever occurred first. The end of follow-up was December 31, 2018. The crude cumulative incidence of all-cause mortality was calculated using the Kaplan-Meier method. The Aalen-Johansen estimator was used to estimate the crude cumulative incidence of heart failure hospitalization and endocarditis while accounting for the competing risk of death. Age- and sex-adjusted incidence rates were obtained using a Poisson model.

The standardized cumulative survival and differences in survival were estimated using flexible parametric regression standardization to account for baseline differences between the groups. The resulting survival curve estimates the population outcome if the entire population either received or did not receive a permanent pacemaker implantation. This method adjusts for the population distribution of covariates.^16^^,^^17^ Flexible hazard-based regression standardization was used to estimate the cumulative incidence and differences in heart failure hospitalization and endocarditis, as described by Kipourou et al.^18^ The produced curves estimate the cumulative incidence of heart failure hospitalization and endocarditis if the entire population either received or did not receive a permanent pacemaker implantation. This method adjusts for the population distribution of covariates while accounting for the competing risk of death. Model selections for all-cause mortality, heart failure hospitalization, and endocarditis were performed using clinical subject matter knowledge and a backward selection strategy aided by the Akaike information criterion. The CART (Classification And Regression Tree) estimation and imputation approach^19^ was used to handle missing data. Data were assumed to be missing at random.

We repeated the main analyses using inverse probability of treatment weighting (IPTW). Propensity scores were generated using generalized boosted regression modeling and stabilized weights were calculated for IPTW to account for differences in baseline characteristics between the pacemaker and non-pacemaker groups.^20^ The main analyses were also repeated in a subset of patients who underwent isolated SAVR. All statistical analyses and data management were performed using the R programming language, version 4.2.0 (R Foundation for Statistical Computing) and included the use of the “survival,” “mexhaz,” and “rstpm2” packages.^21^, ^22^, ^23^

### Missing data

Missing data were present in the following variables: left ventricular ejection fraction (9.6%), body mass index (7.3%), preoperative dialysis (5.4%), estimated glomerular filtration rate (2.9%), education level (0.9%), and valve size (0.8%).

## Results

In total, 19,576 patients underwent SAVR with low surgical risk in Sweden from 2001 to 2018 and fulfilled the inclusion criteria. Patients had a mean age of 68 ± 12 years and included 13,093 men (67%) and 6,483 women (33%). There were small, but potentially important, baseline differences between the groups: for example, prior heart failure (20% in the pacemaker group vs 16% in the no pacemaker group) and left ventricular ejection fraction <30% (4.5% in the pacemaker group vs 1.8% in the no pacemaker group). Baseline characteristics are presented in Table 1. The rate of permanent pacemaker implantations per year increased during the study period, as shown in Supplemental Figure 1. The distribution of pacemaker implantations within 30 days following SAVR is shown in Supplemental Figure 11.

**Table 1:** Baseline Characteristics of Patients With and Without Permanent Pacemaker Implantation After Surgical Aortic Valve Replacement Between 2001 and 2018 in Sweden

	Overall (N = 19,576)	Pacemaker (n = 732, 3.7%)	No Pacemaker (n = 18,844, 96.3%)
Age, y	67.6 ± 11.5	67.1 ± 12.1	67.6 ± 11.4
Male	13,093 (66.9)	488 (66.7)	12,605 (66.9)
Non-Nordic birth region	1,267 (6.5)	50 (6.8)	1,217 (6.5)
Education level			
<10 y	7,718 (39.8)	276 (38.1)	7,442 (39.9)
10–12 y	7,732 (39.9)	308 (42.5)	7,424 (39.8)
>12 y	3,948 (20.4)	141 (19.4)	3,807 (20.4)
Household income			
Q1 (lowest)	4,894 (25.0)	183 (25.0)	4,711 (25.0)
Q2	4,894 (25.0)	179 (24.5)	4,715 (25.0)
Q3	4,894 (25.0)	176 (24.0)	4,718 (25.0)
Q4 (highest)	4,893 (25.0)	194 (26.5)	4,699 (24.9)
Married	12,159 (62.1)	431 (58.9)	11,728 (62.2)
Body mass index, kg/m^2^			
<18.5	141 (0.8)	7 (1.0)	134 (0.8)
18.5–24.9	5,766 (31.8)	231 (34.5)	5,535 (31.7)
25-29.9	7,882 (43.4)	275 (41.0)	7,607 (43.5)
≥30	4,360 (24.0)	157 (23.4)	4,203 (24.0)
Prior atrial fibrillation	2,815 (14.4)	120 (16.4)	2,695 (14.3)
Prior heart failure	3,177 (16.2)	147 (20.1)	3,030 (16.1)
LVEF			
>50%	14,393 (81.3)	521 (76.4)	13,872 (81.5)
30–50%	2,974 (16.8)	130 (19.1)	2,844 (16.7)
<30%	337 (1.9)	31 (4.5)	306 (1.8)
COPD	1,484 (7.6)	38 (5.2)	1,446 (7.7)
Diabetes mellitus	3,584 (18.3)	136 (18.6)	3,448 (18.3)
eGFR, mL/min/1.73 m^2^			
≥60	15,067 (79.3)	552 (78.9)	14,515 (79.3)
45–59	2,757 (14.5)	103 (14.7)	2,654 (14.5)
30–44	902 (4.7)	33 (4.7)	869 (4.7)
<30	279 (1.5)	12 (1.7)	267 (1.5)
Preoperative dialysis	117 (0.6)	3 (0.4)	114 (0.6)
Prior myocardial infarction	2,175 (11.1)	85 (11.6)	2,090 (11.1)
Prior PCI	1,337 (6.8)	45 (6.1)	1,292 (6.9)
Peripheral vascular disease	1,914 (9.8)	73 (10.0)	1,841 (9.8)
Hypertension	9,319 (47.6)	356 (48.6)	8,963 (47.6)
Hyperlipidemia	3,937 (20.1)	134 (18.3)	3,803 (20.2)
Prior stroke	1,770 (9.0)	70 (9.6)	1,700 (9.0)
History of cancer	2,637 (13.5)	107 (14.6)	2,530 (13.4)
Alcohol dependence	524 (2.7)	11 (1.5)	513 (2.7)
Hepatic disease	263 (1.3)	5 (0.7)	258 (1.4)
Prior major bleeding event	1,359 (6.9)	42 (5.7)	1,317 (7.0)
Isolated AVR	11,840 (60.5)	480 (65.6)	11,360 (60.3)
Concomitant CABG	5,998 (30.6)	182 (24.9)	5,816 (30.9)
Ascending aortic surgery	1,951 (10.0)	72 (9.8)	1,879 (10.0)
Valve size, mm			
18-21	5,678 (29.2)	186 (25.8)	5,492 (29.4)
22-23	7,480 (38.5)	280 (38.8)	7,200 (38.5)
24-29	6,261 (32.2)	256 (35.5)	6,005 (32.1)
Period of surgery, y			
2001-2008	7,768 (39.7)	251 (34.3)	7,517 (39.9)
2009-2013	5,737 (29.3)	207 (28.3)	5,530 (29.3)
2014-2018	6,071 (31.0)	274 (37.4)	5,797 (30.8)
Bioprosthesis	14,331 (73.2)	522 (71.3)	13,809 (73.3)

Values are mean ± SD or n (%).

CABG = coronary artery bypass grafting; COPD = chronic obstructive pulmonary disease; eGFR = estimated glomerular filtration rate; LVEF = left ventricular ejection fraction; PCI = percutaneous coronary intervention.

### Clinical outcomes

Table 2 shows the regression standardized cumulative incidences and differences for all outcomes at 5, 10, 15, and 17 years after surgical treatment. The crude cumulative incidences for all outcomes are shown in Supplemental Table 2. The crude and age- and sex-adjusted incidence rates for all outcomes are shown in Supplemental Table 3.

**Table 2:** Regression Standardized Cumulative Incidence and Differences for All-Cause Mortality, Heart Failure Hospitalization, and Endocarditis Among Patients With Low Surgical Risk Who Underwent Surgical Aortic Valve Replacement in Sweden

	5 Years	10 Years	15 Years	17 Years
All-cause mortality				
Pacemaker	12 (10-13)	29 (26-31)	47 (43-50)	53 (49-57)
No pacemaker	12 (11-12)	29 (28-29)	46 (45-48)	53 (51-54)
Difference	0 (−1.4 to 1.5)	0.1 (−2.9 to 3.0)	0.1 (−3.6 to 3.7)	0.1 (−3.6 to 3.8)
Heart failure hospitalization				
Pacemaker	7.5 (6.3-9.1)	17 (14-20)	25 (22-30)	28 (24-33)
No pacemaker	5.1 (4.7-5.4)	12 (11-12)	18 (17-19)	20 (19-22)
Difference	2.5 (1.1-3.9)	5.3 (2.4-8.2)	7.5 (3.4-12)	8.2 (3.8-13)
Endocarditis				
Pacemaker	3.3 (2.3-4.7)	5.6 (3.9-7.9)	7.6 (5.3-11)	7.9 (5.5-11)
No pacemaker	3.0 (2.8-3.3)	5.2 (4.7-5.6)	7.0 (6.3-7.8)	7.3 (6.6-8.2)
Difference	0.3 (−0.9 to 1.4)	0.4 (−1.6 to 2.4)	0.6 (−2.1 to 3.2)	0.6 (−2.2 to 3.3)

Values are % (95% CI). Adjusted by regression standardization. Model covariates included were postoperative permanent pacemaker implantation, age, sex, hospital, left ventricular ejection fraction, concomitant coronary artery bypass, ascending aortic surgery, birth region, education level, prior atrial fibrillation, history of cancer, diabetes mellitus, prior endocarditis, prior heart failure, hyperlipidemia, hypertension, hepatic disease, prior peripheral vascular disease, prior stroke, prior major bleeding event, prior percutaneous coronary intervention, household income, categorical body mass index, period of surgery, categorical estimated glomerular filtration rate, categorical valve size, isolated AVR, and bioprothesis. A detailed description and precise model specification for the different outcomes is available in the Supplemental Appendix.

### All-cause mortality

During a mean follow-up of 7.6 years (maximum 19.2 years), 5,848 patients (30%) died.

**Figure 1:**
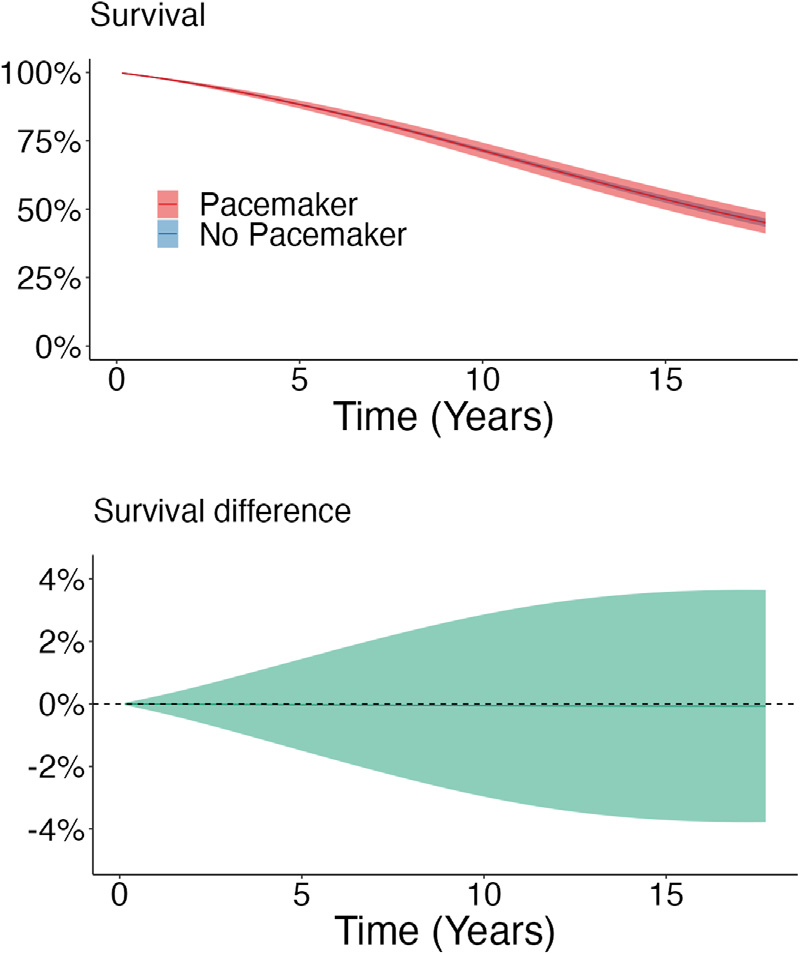
**Regression Standardized Survival and Difference in Survival** (Upper panel) The curves represent the estimated survival and 95% CI if the population either had received permanent pacemaker implantation or had not received permanent pacemaker implantation, respectively. For example, if the entire population had received a permanent pacemaker implantation, the estimated population survival at 17 years would be 47%. (Lower panel) Estimated difference in survival (95% CI) between the pacemaker and no pacemaker groups.

After 17 years of follow-up, the regression standardized cumulative incidence for all-cause mortality was 53% (95% CI: 49%-57%) in the pacemaker group compared to 53% (95% CI: 51%-54%) in the no pacemaker group, with a 0.1% (95% CI: -3.6% to 3.8%) difference between the groups (Table 2). The regression standardized cumulative survival is shown in Figure 1.

### Heart failure hospitalization

**Figure 2:**
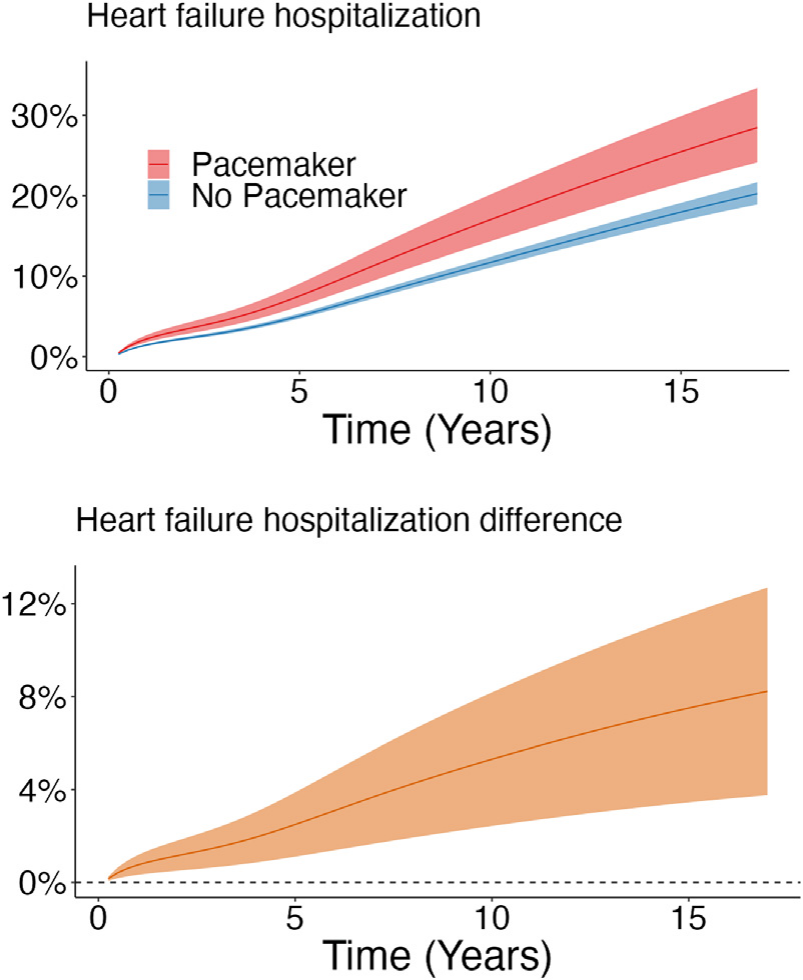
**Regression Standardized Heart Failure Hospitalization and Difference in Heart Failure Hospitalization** (Upper panel) The curves represent the estimated cumulative incidence of heart failure hospitalization and 95% CI if the population either had received permanent pacemaker implantation or had not received permanent pacemaker implantation, respectively. for example, if the entire population had received a permanent pacemaker, the estimated population cumulative incidence of heart failure hospitalization at 17 years would be 28%. (Lower panel) Estimated difference in cumulative incidence of heart failure hospitalization (95% CI) between the patients who had received permanent pacemaker implantation and those who had not.

During a mean follow-up of 6.5 years (maximum 18.0 years), 2,113 patients (10.8%) were hospitalized for heart failure. After 17 years of follow-up, the regression standardized cumulative incidence for heart failure hospitalization was 28% (95% CI: 24%-33%) in the pacemaker group compared to 20% (95% CI: 19%-22%) in the no pacemaker group, with an 8.2% (95% CI: 3.8%-13%) difference between the groups (Table 2; Central Illustration). The regression standardized cumulative incidence of heart failure hospitalization for both groups is shown in Figure 2.

**Central Illustration:**
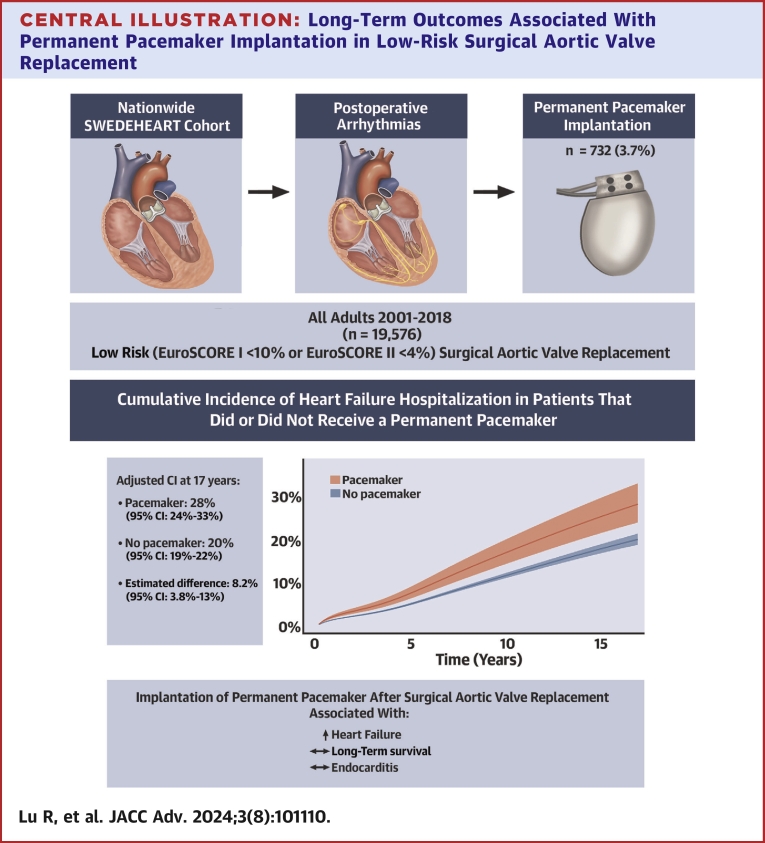
**Long-Term Outcomes Associated With Permanent Pacemaker Implantation in Low-Risk Surgical Aortic Valve Replacement** Permanent pacemaker implantation after surgical aortic valve replacement in patients with low surgical risk was associated with an increased risk of heart failure in a nationwide cohort study including 19,576 patients operated between 2001 and 2018 in Sweden.

### Endocarditis

**Figure 3:**
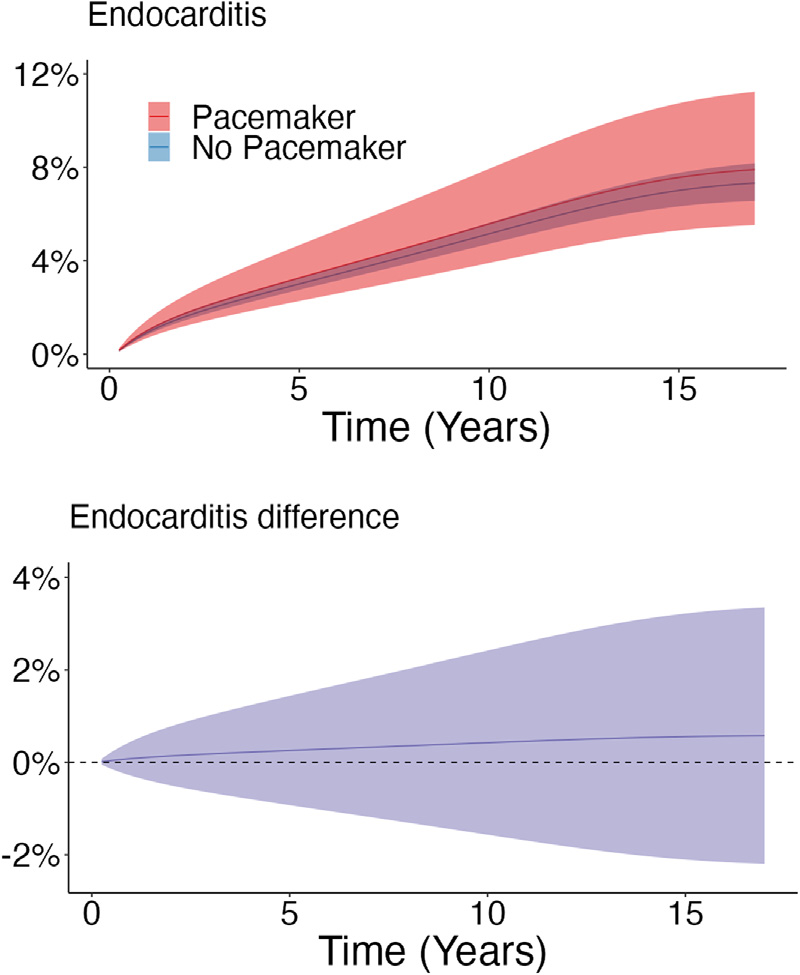
**Regression Standardized Endocarditis and Difference in Endocarditis** (Upper panel) The curves represent the estimated cumulative incidence of endocarditis and 95% CI if the population either had received permanent pacemaker implantation or had not received permanent pacemaker implantation, respectively. For example, if the entire population had received a permanent pacemaker implantation, the estimated population cumulative incidence of endocarditis at 17 years would be 7.9%. (Lower panel) Estimated difference in cumulative incidence of endocarditis (95% CI) between the pacemaker and no pacemaker groups.

During a mean follow-up of 6.6 years (maximum 18.0 years), 814 patients (4.2%) had endocarditis. After 17 years of follow-up, the regression standardized cumulative incidence for endocarditis was 7.9% (95% CI: 5.5%-11%) in the pacemaker group compared to 7.3% (95% CI: 6.6%-8.2%) in the no pacemaker group with a 0.6% (95% CI: -2.2% to 3.3%) difference between the groups (Table 2). The regression standardized cumulative incidence of endocarditis for both groups is shown in Figure 3.

### Sensitivity analyses

The findings of the sensitivity analyses conducted on the subset of patients who underwent isolated SAVR (Supplemental Figures 2 to 4) and the analyses utilizing IPTW (Supplemental Figures 5 to 7) were consistent with the main analyses.

## Discussion

In patients with low surgical risk, permanent pacemaker implantation after SAVR was associated with a higher risk of heart failure hospitalization compared to no permanent pacemaker implantation. There was no association between permanent pacemaker implantation and all-cause mortality, or endocarditis.

Right ventricular pacing has been associated with an increased risk of heart failure. A study that included all 27,704 patients without prior heart failure who received a right ventricle lead in Denmark between 2002 and 2014 investigated the risk of heart failure compared to an age- and sex-matched control population.^24^ They found a higher risk for heart failure in patients with a right ventricle lead (HR: 1.11 [95% CI: 1.04-1.17]).^24^ While the mechanism is not fully understood, it is likely that the dyssynchronous activation pattern in right ventricle pacing leads to adverse remodeling and decreased pumping ability of the ventricles.^25^

In a previous study from our group, the prognosis after permanent pacemaker implantation following SAVR was analyzed among 24,983 patients, who underwent SAVR in Sweden from 1997 to 2018 irrespective of surgical risk.^1^ In this study, 3.4% of patients received a permanent pacemaker and the absolute risk difference for heart failure between patients with permanent pacemaker implantation and no pacemaker was 9.6% (95% CI: 4.9%-14.2%) after 15 years of follow-up. These results are similar to our study, where we found an absolute risk difference between pacemaker and no pacemaker of 7.5% (95% CI: 3.4%-12%) after 15 years. In contrast, the prior study also found a significant association between permanent pacemaker and all-cause mortality with an absolute risk difference of 4.9% at 15 years (95% CI: 0.5%-9.2%), while the current study showed a nonsignificant absolute risk difference of 0.1% (95% CI: -3.6% to 3.7%). It is possible that permanent pacemaker requires a certain burden of comorbidity to translate into increased mortality. Among patients with low surgical risk, this burden of comorbidity might not be sufficient to increase the mortality rate in patients with permanent pacemaker. Similar to the prior study, we found no association between permanent pacemaker implantation after SAVR and endocarditis.

Although the current study constitutes a subgroup of patients from our prior publication that included patients irrespective of surgical risk,^1^ we believe this study provides results and data that are novel, unique, and important. The solely inclusion of patients with low surgical risk in this study provides relevant information for this specific group which, to the best of our knowledge, is nonexistent today. In the current study, the inclusion of patients operated from 2001 onward yields a more contemporary time-period compared with a study period from 1997, as in the previous study.^1^

In another study, Greason et al^26^ investigated the association between permanent pacemaker implantation after SAVR and all-cause mortality in 5,842 patients operated at the Mayo Clinic (Rochester, Minnesota) between 1993 and 2014, irrespective of surgical risk. In their study, 2.5% of the patients received a permanent pacemaker and the study population had a median Society of Thoracic Surgeons risk score of 3% similar to the study by Glaser et al.^1^ They also found a significant association between permanent pacemaker implantation and long-term mortality (HR: 1.49; 95% CI: 1.20-1.84).^26^

A study by Rück and colleagues^4^ investigated outcomes after permanent pacemaker implantation following TAVR in a nationwide study from Sweden between 2008 and 2018. Of 3,420 patients included, 14% underwent permanent pacemaker implantation. They found no difference in all-cause mortality, cardiovascular mortality, heart failure, or endocarditis between patients with and without permanent pacemaker. The authors discussed that although they had a maximum follow-up of 11.8 years (median 2.7 years), longer follow-up might be needed to detect a difference in survival and heart failure. More research on the effect of pacemaker implantation in patients with low surgical risk following TAVR is needed.

While it is reassuring that neither mortality nor endocarditis is associated with permanent pacemaker in patients with low surgical risk following SAVR, the increased incidence of heart failure gives reason to act. Apart from a careful and meticulous technique during surgery, options to reduce the risk of permanent pacemaker might include the avoidance of valves associated with an increased risk, excessive sizing, and the choice of SAVR over TAVR.^4^^,^^27^ However, steps to avoid potentially unnecessary use of permanent pacemakers might have a greater impact on minimizing adverse outcomes related to permanent pacemaker implantation than the pacemaker itself, and therefore requires careful consideration.

The ideal timing of permanent pacemaker implantation after surgery remains controversial. Some studies have found that only 40% to 45% of patients who had received permanent pacemaker implantation were dependent on pacemaker in the long-term.^28^^,^^29^ This suggests that some perioperative injuries of the heart conduction system may recover over time.^29^ While there is a clear indication for early permanent pacemaker implantation in patients with complete AV block with low or no escape rhythm following SAVR,^30^ other perioperative injuries may be transient and resolve spontaneously.^28^^,^^29^ Consequently, the optimal timing of permanent pacemaker implantation in patients with less severe conduction disturbances may warrant further research. While a delay in permanent pacemaker implantation might reduce unnecessary use, other factors such as risks associated with prolonged hospital stay and health economics must be taken into consideration.

Our study is clinically relevant, especially in an era where more young patients with low surgical risk become subject to TAVR procedures. Younger patients with low surgical risk have a longer life expectancy,^31^ making adverse outcomes associated with permanent pacemaker implantation very relevant for this population in terms of quality-adjusted life years.

### Strengths and limitations

The inclusion of all patients who underwent SAVR at all hospitals performing cardiac surgery in Sweden resulted in a large study population, with a high degree of generalizability. Information from several high-quality and complete nationwide health data registers in Sweden was linked, which allowed for careful characterization of the study population, including demographics, medical history, comorbidities, and socioeconomic status. Access to these data made adjustment for a wide range of potential confounders possible.

Our study had some limitations. First, since this was an observational study, residual confounding might be present. Second, although the amount of missing baseline data was low, it could potentially have influenced the results if not missing completely at random. Despite detailed characterization of the study population, our database did not include potentially important patient features, such as information about the electrocardiogram, pacing indications, and right ventricular pacing percentages at follow-up. Third, we were not able to ascertain the outcomes of heart failure hospitalization and endocarditis that occurred outside of Sweden. However, due to the universal tax-financed health care coverage in Sweden, these number of patients were likely minimal. All deaths that occurred abroad were captured by the Population Register, and follow-up for death was therefore complete.^9^

## Conclusions

We found an increased incidence of heart failure in patients with low surgical risk who received a permanent pacemaker after SAVR. No association was observed between permanent pacemaker implantation and long-term all-cause mortality, or risk of endocarditis. Clinicians should be aware of the potential risks associated with pacemaker implantation in this patient population. Efforts should be made to minimize the requirement for permanent pacemaker implantation following SAVR.

Perspectives **COMPETENCY IN MEDICAL KNOWLEDGE**: Clinicians are urged to exercise thorough consideration prior to making decisions regarding permanent pacemaker implantation in low surgical risk patients. Efforts should be taken to minimize the necessity for permanent pacemaker implantation after SAVR.

**TRANSLATIONAL OUTLOOK:** Patients with low surgical risk who received a permanent pacemaker after SAVR had a higher risk of heart failure. Future investigations should prioritize examining the optimal timing for permanent pacemaker implantation after SAVR, a matter that remains controversial. Given the observed association of pacemaker implantation with adverse outcomes, a careful consideration of strategies aimed at their avoidance is needed.

## Funding support and author disclosures

This work was supported by the 10.13039/501100003793Swedish Heart-Lung Foundation (grant number 20190533 to Dr Sartipy and grant number 20190570 to Dr Glaser), Region Stockholm (ALF Project) (grant number FoUI-962048 to Dr Sartipy and grant number FoUI-954783 and FoUI-961871 to Dr Glaser), Region Stockholm clinical postdoctoral appointment (FoUI-955489 to Dr Glaser), the 10.13039/501100007687Swedish Society of Medicine (grant number SLS-934749 to Dr Glaser), the Eva and Oscar Ahrén Research Foundation (to Dr Glaser), the 10.13039/100018876Seraphim Hospital Foundation (to Dr Glaser), 10.13039/501100006285Magnus Bergvall Foundation (grant number 2021-04333 and grant number 2022-117 to Dr Glaser), Mats Kleberg Foundation (grant number 2022-119 to Dr Glaser), 10.13039/501100004047Karolinska Institutet Foundations and Funds (grant number 2022-01575 to Dr Glaser), donations from the Schörling Foundation, and Mr Fredrik Lundberg. The authors have reported that they have no relationships relevant to the contents of this paper to disclose.
