# Supplementary material for: Long-Term Outcomes Associated With Permanent Pacemaker Implantation in Low-Risk Surgical Aortic Valve Replacement
Source: JACC Adv. 2024 Jul 18;3(8):101110. doi: 10.1016/j.jacadv.2024.101110 (PMC11293502; doi:10.1016/j.jacadv.2024.101110)

**SUPPLEMENTAL APPENDIX**

# Supplemental Methods: Statistical methods

### Regression standardization

Regression standardization can be used to obtain an adjusted survival curve, that can be helpful to describe the population effect of a treatment. The method can be simply described by the following steps. First, model the outcome with exposure and potential covariates. Then, given a binary exposure, the population dataset is copied, with each copy the exposure variable is set to one level of the exposure among all individuals in that copied dataset. The initial model is then used to predict the outcome for each of the patients in each copy of the population, and then average these predictions to get a population estimate. In the case of a binary exposure and a survival outcome, this method can be used to yield two survival curves, one for exposed, and one for non-exposed. Each curve represent the hypothetical population where all patients either received, or did not receive the treatment, standardized by (or adjusted for) the population distribution of covariates. The results are intuitive and can be interpreted as if the entire population received treatment X, 70% would be alive at 10 years, and if the entire population received treatment Y, only 50% would be alive at 10 years. Regression standardization answers what in causal inference is referred to as a counterfactual question, characterized by its “if instead…” nature. For further reading we recommend, Sjölander 2016, Kipouro 2019 and Rothman 2012 p.475-478, 518-521 ^1–3^.

### Model selection

The model selection strategy combined subject matter knowledge and backward selection using all variables in Table 1. The final model selection was informed using the Akaike information criterion (AIC). Continuous covariates were tested using splines and polynomials.

### Survival

### The baseline hazard was modeled using a natural spline with three degrees of freedom.

### Age was centered and included as a quadratic term. Splines of age did not improve model performance. The covariates included in the final model were: postoperative permanent pacemaker implantation, age, sex, hospital, left ventricular ejection fraction, concomitant coronary artery bypass, ascending aortic surgery, birth region, education level, prior atrial fibrillation, alcohol dependence, prior atrial fibrillation, history of cancer, chronic obstructive pulmonary disease, diabetes mellitus, prior endocarditis, prior heart failure, hyperlipidemia, hypertension, hepatic disease, prior peripheral vascular disease, prior stroke, prior major bleeding event, marital status, prior percutaneous coronary intervention, household income, categorical body mass index, period of surgery, categorical estimated glomerular filtration rate, categorical valve size, and bioprothesis.

### Heart failure hospitalization

### The final model for heart failure hospitalization used a 3rd degree exponential b-spline with 2 inner knots at the 1/3 and 2/3 quantiles for the baseline hazard. Age was centered and included as a quadratic term. Splines of age did not improve model performance. The covariates included were: postoperative permanent pacemaker implantation, age, sex, hospital, left ventricular ejection fraction, concomitant coronary artery bypass, ascending aortic surgery, birth region, education level, prior atrial fibrillation, alcohol dependence, prior atrial fibrillation, history of cancer, chronic obstructive pulmonary disease, diabetes mellitus, prior endocarditis, prior heart failure, hypertension, hepatic disease, prior stroke, prior major bleeding event, prior percutaneous coronary intervention, household income, categorical body mass index, period of surgery, categorical estimated glomerular filtration rate, categorical valve size, isolated AVR, and bioprothesis.

### Endocarditis

### The final model for endocarditis used a 3rd degree exponential b-spline with 2 inner knots at the 1/3 and 2/3 quantiles for the baseline hazard. Age was centered and included as a quadratic term. Splines of age did not improve model performance. The covariates included were: postoperative permanent pacemaker implantation, age, sex, hospital, left ventricular ejection fraction, concomitant coronary artery bypass, ascending aortic surgery, birth region, education level, prior atrial fibrillation, history of cancer, diabetes mellitus, prior endocarditis, prior heart failure, hyperlipidemia, hypertension, hepatic disease, prior peripheral vascular disease, prior stroke, prior major bleeding event, prior percutaneous coronary intervention, household income, categorical body mass index, period of surgery, categorical estimated glomerular filtration rate, categorical valve size, isolated AVR, and bioprothesis.

## Supplemental References

1. Sjölander A. Regression standardization with the R package stdReg. *Eur J Epidemiol*. 2016;31:563–574.

2. Kipourou D, Charvat H, Rachet B, Belot A. Estimation of the adjusted cause‐specific cumulative probability using flexible regression models for the cause‐specific hazards. *Statistics in Medicine*. 2019;38:3896–3910.

3. Rothman KJ, Lash TL, VanderWeele TJ, Haneuse S. *Modern epidemiology*. 4th edition. Philadelphia: Wolters Kluwer, 2021.

| Supplemental Table 1. ICD codes used to identify secondary outcomes and exposure in patients who underwent aortic valve replacement in Sweden between 2001 and 2018. | |
| --- | --- |
| Outcome and exposure | ICD9-10 codes |
| Heart Failure Hospitalization | I50, I42, I43, I25.5,  K761, I11.0, I13.0, 425, 428. |
| Endocarditis | I33.0, 133.9, I38.9, I39.8, 421 |
| Permanent Pacemaker Implantation | FPE00, FPE10, FPE20, FPE26, FPF00,  FPF10, FPF20, FPG10, FPG20, FPG30, FPG33. |
| ICD = International Classification of Diseases | |

| Supplemental Table 2. Crude cumulative incidence for all-cause mortality, heart failure hospitalization and endocarditis. % (95% CI) | | | | |
| --- | --- | --- | --- | --- |
|  | 5 years | 10 years | 15 years | 17 years |
| All-cause mortality | | | | |
| Pacemaker | 12 (9-15) | 34 (30-39) | 53 (47-60) | 58 (49-66) |
| No Pacemaker | 12 (12-13) | 32 (32-33) | 54 (52-55) | 60 (59-62) |
| Heart failure hospitalization | | | | |
| Pacemaker | 11 (8.0-13) | 19 (16-23) | 25 (20-30) | 25 (20-30) |
| No Pacemaker | 5.6 (5.3-6.0) | 13 (12-14) | 20 (19-21) | 22 (21-23) |
| Endocarditis | | | | |
| Pacemaker | 3.2 (1.8-4.7) | 6.1 (3.7-8.4) | 6.1 (3.7-8.4) | 7.4 (3.9-11) |
| No Pacemaker | 3.1 (2.9-3.4) | 5.0 (4.6-5.4) | 6.4 (5.9-6.9) | 6.6 (6.1-7.2) |
| CI = confidence interval. Heart failure rehospitalization and endocarditis using Aalen-Johansen estimator accounting for the competing risk of death. | | | | |

| Supplemental Table 3. Incidence rates per 100 person-years of all-cause mortality, heart failure hospitalization and endocarditis after surgical aortic valve replacement between 2001 to 2018 in Sweden, in low-risk patients. | | |
| --- | --- | --- |
| Outcome | Pacemaker | No Pacemaker |
| All-cause mortality | | |
| No. of events | 199 | 5649 |
| Crude | 3.9 (3.3-4.4) | 3.9 (3.8-4.0) |
| Age- and sex-adjusted | 4.2 (4.1-4.3) | 4.2 (3.7-4.7) |
| Heart failure hospitalization | | |
| No. of events | 103 | 2010 |
| Crude | 2.5 (2.0-3.0) | 1.6 (1.6-1.7) |
| Age- and sex-adjusted | 2.7 (2.5-2.8) | 1.7 (1.4-2.1) |
| Endocarditis | | |
| No. of events | 29 | 785 |
| Crude | 0.7 (0.4-0.9) | 0.6 (0.6-0.7) |
| Age- and sex-adjusted | 0.6 (0.6-0.7) | 0.6 (0.4-0.9) |
| Age- and sex-adjusted incidence rates were obtained from a Poisson model. CI = confidence interval. | | |

Supplemental Figure 1. Proportion of operated patients who received permanent pacemaker implantation among patients with low surgical risk per year, in Sweden from 2001 to 2018.


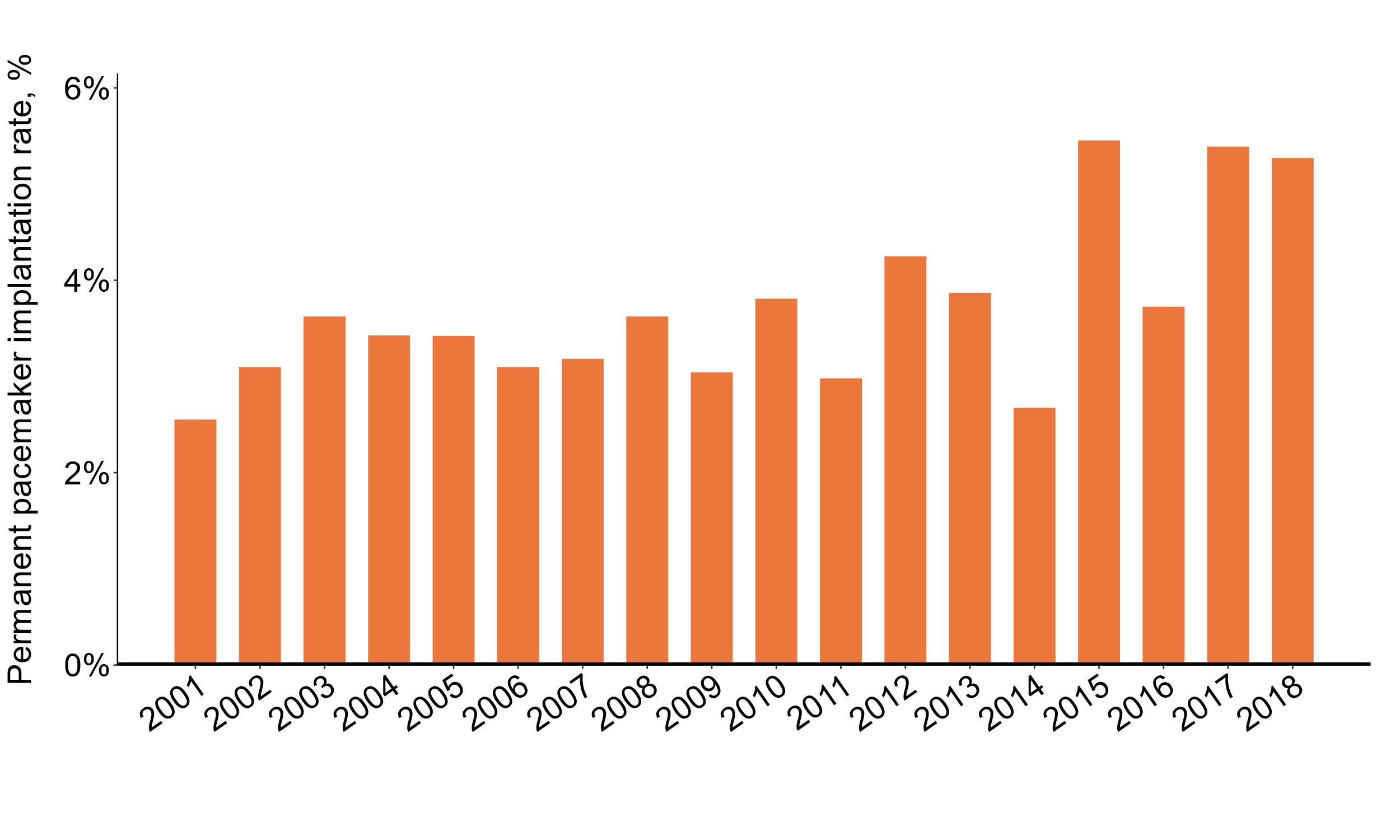


Supplemental Figure 2. Regression standardized survival and survival difference in the subgroup population that underwent isolated surgical AVR**.**

**Upper panel:** The curves represent the estimated survival and 95% CI if the population either had received permanent pacemaker implantation or had not received permanent pacemaker implantation, respectively. For example, if the entire population had received a permanent pacemaker implantation, the estimated population survival at 17 years would be 53%. **Lower panel:** Estimated difference in survival (95% CI) between the pacemaker and no pacemaker groups. *CI = confidence interval.*

**
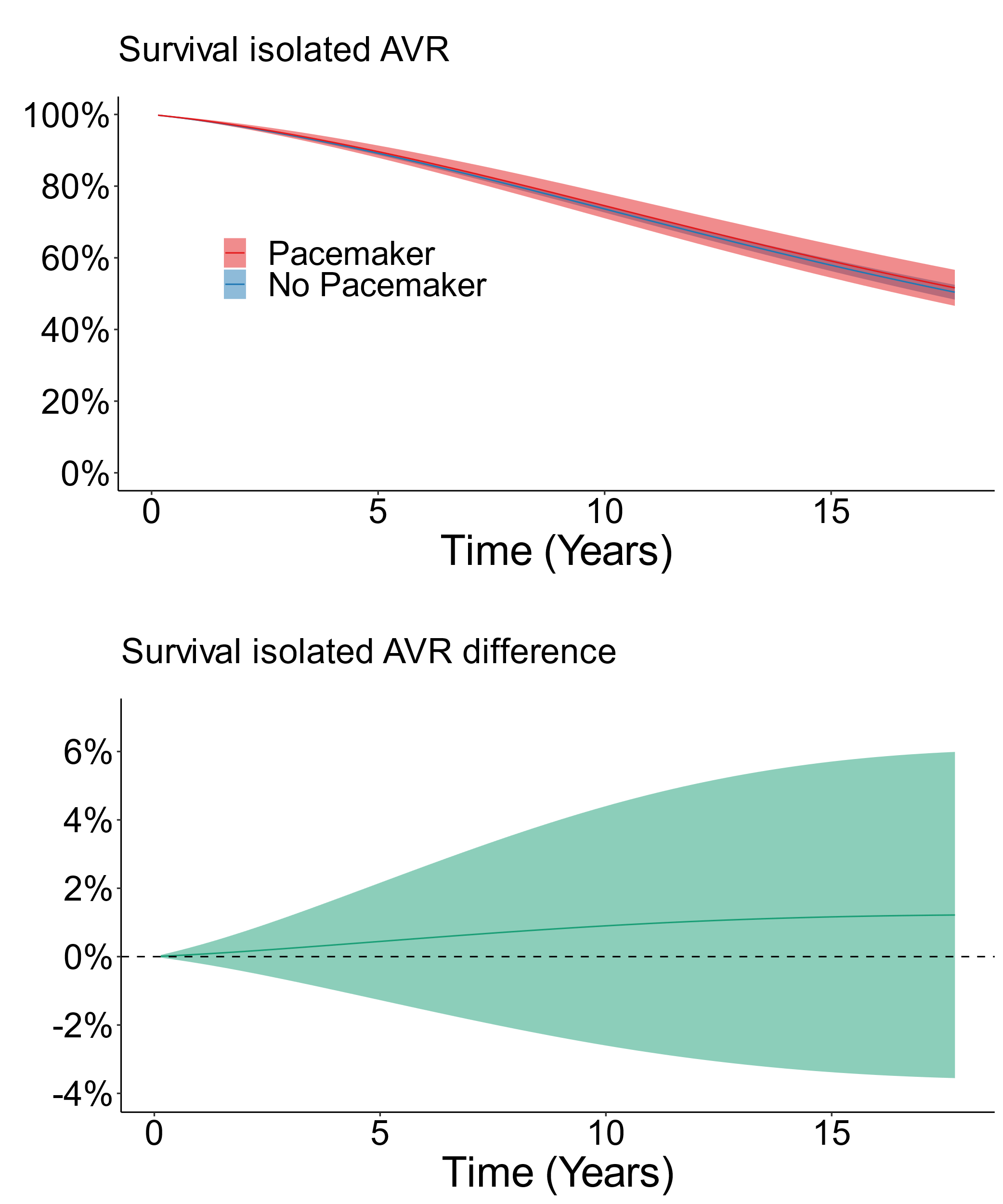
**

Supplemental Figure 3. Regression standardized heart failure hospitalization and heart failure hospitalization difference in the subgroup population that underwent isolated surgical AVR**.**

**Upper panel:** The curves represent the estimated heart failure hospitalization and 95% CI if the entire population had received permanent pacemaker implantation or had not received permanent pacemaker implantation, respectively. For example, if the entire population had received a permanent pacemaker, the estimated population heart failure hospitalization at 17 years would be 24%. **Lower panel:** Estimated difference in heart failure hospitalization (95% CI) between the pacemaker and no pacemaker groups. *CI = confidence interval.*


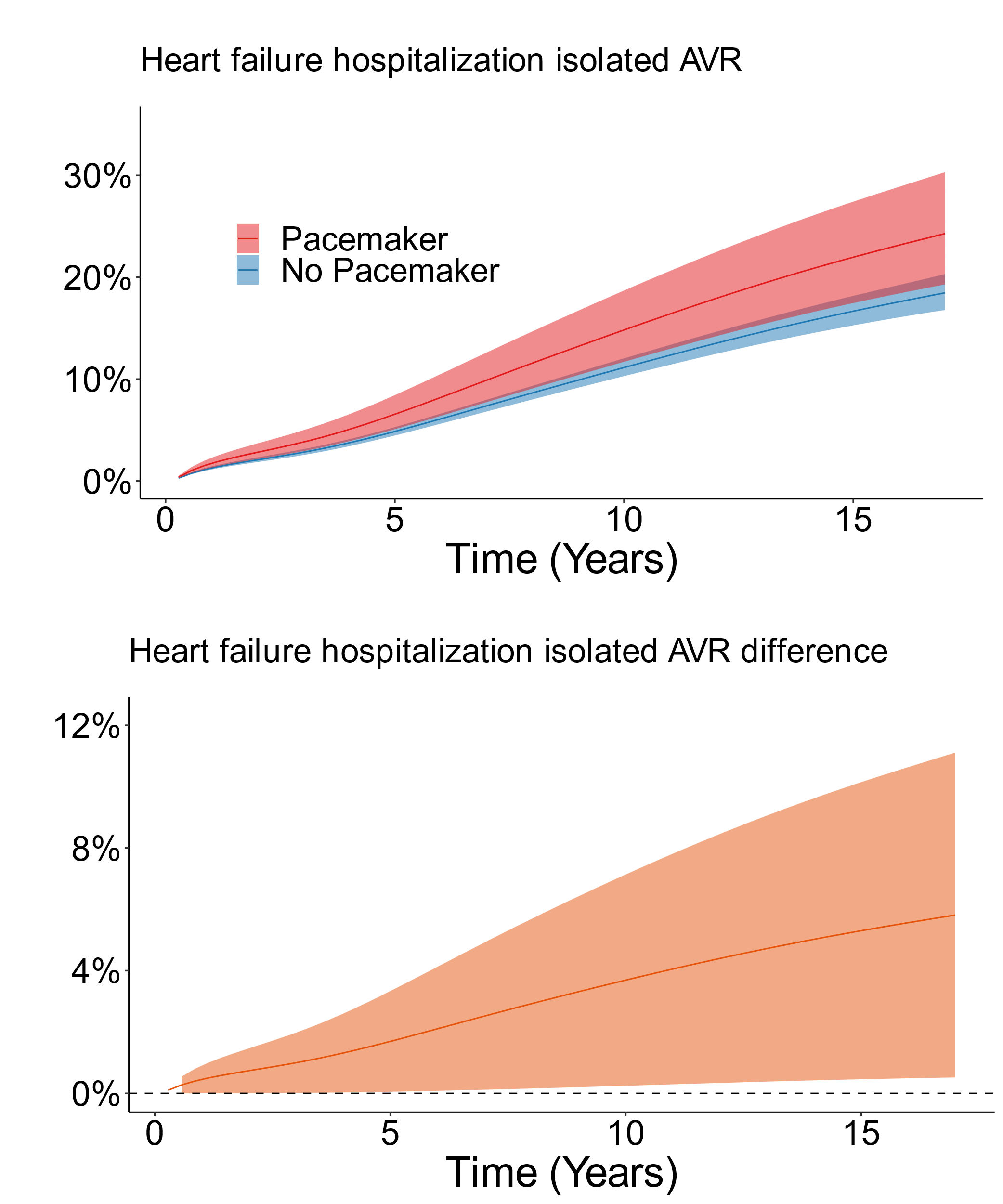


Supplemental Figure 4. Regression standardized endocarditis and endocarditis difference in the subgroup population that underwent isolated surgical AVR**.**

**Upper panel:** The curves represent the estimated cumulative incidence of endocarditis and 95% CI if the population either had received permanent pacemaker implantation or had not received permanent pacemaker implantation, respectively. For example, if the entire population had received a permanent pacemaker implantation, the estimated population endocarditis risk at 17 years would be 6.7%. **Lower panel:** Estimated difference in endocarditis (95% CI) between the pacemaker and no pacemaker groups. *CI = confidence interval*

**
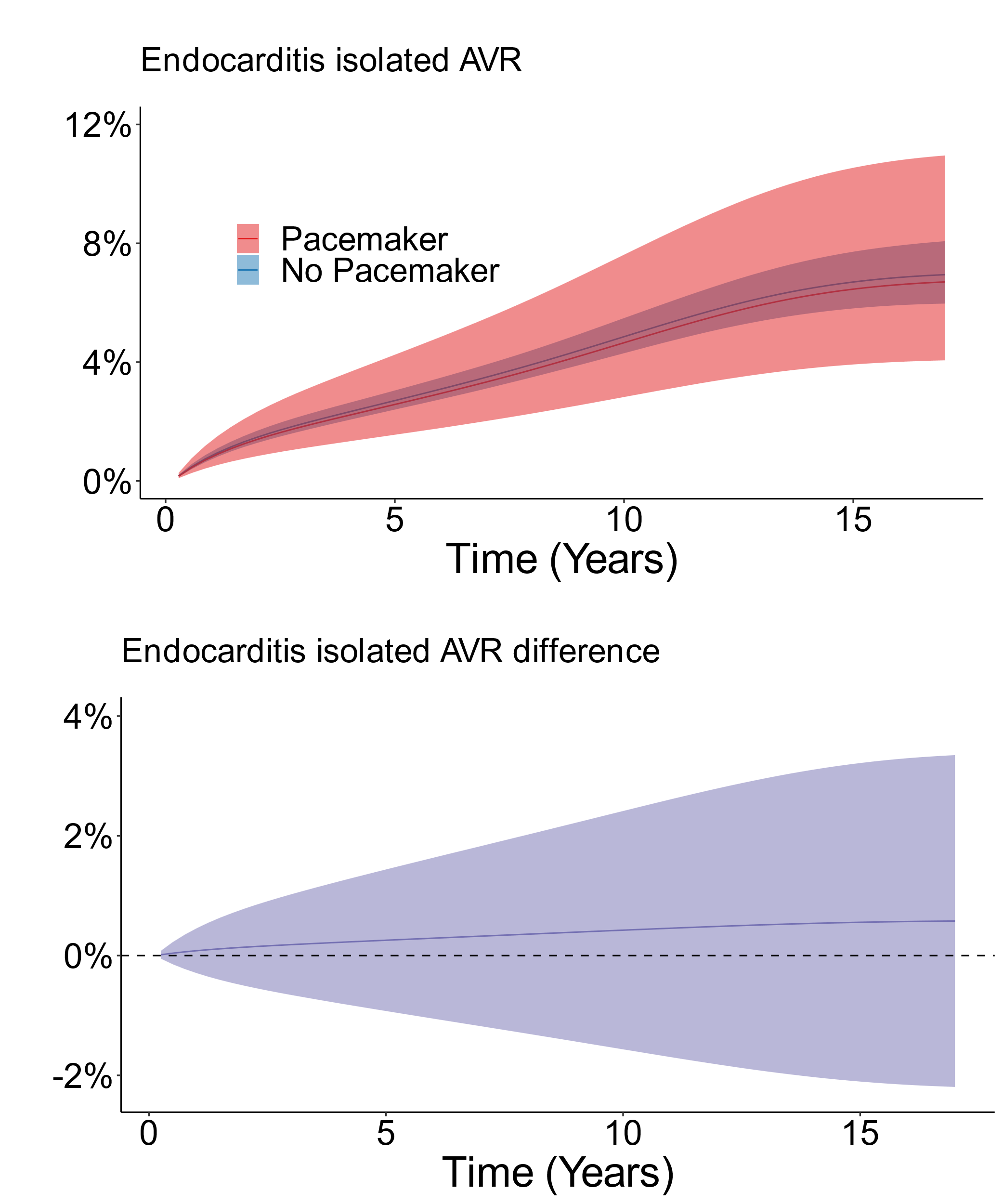
**

Supplemental Figure 5. Kaplan-Meier estimated survival after inverse probability of treatment weighting in patients who received permanent pacemaker implantation and those who did not receive permanent pacemaker implantation after surgical AVR in Sweden between 2001 and 2018.


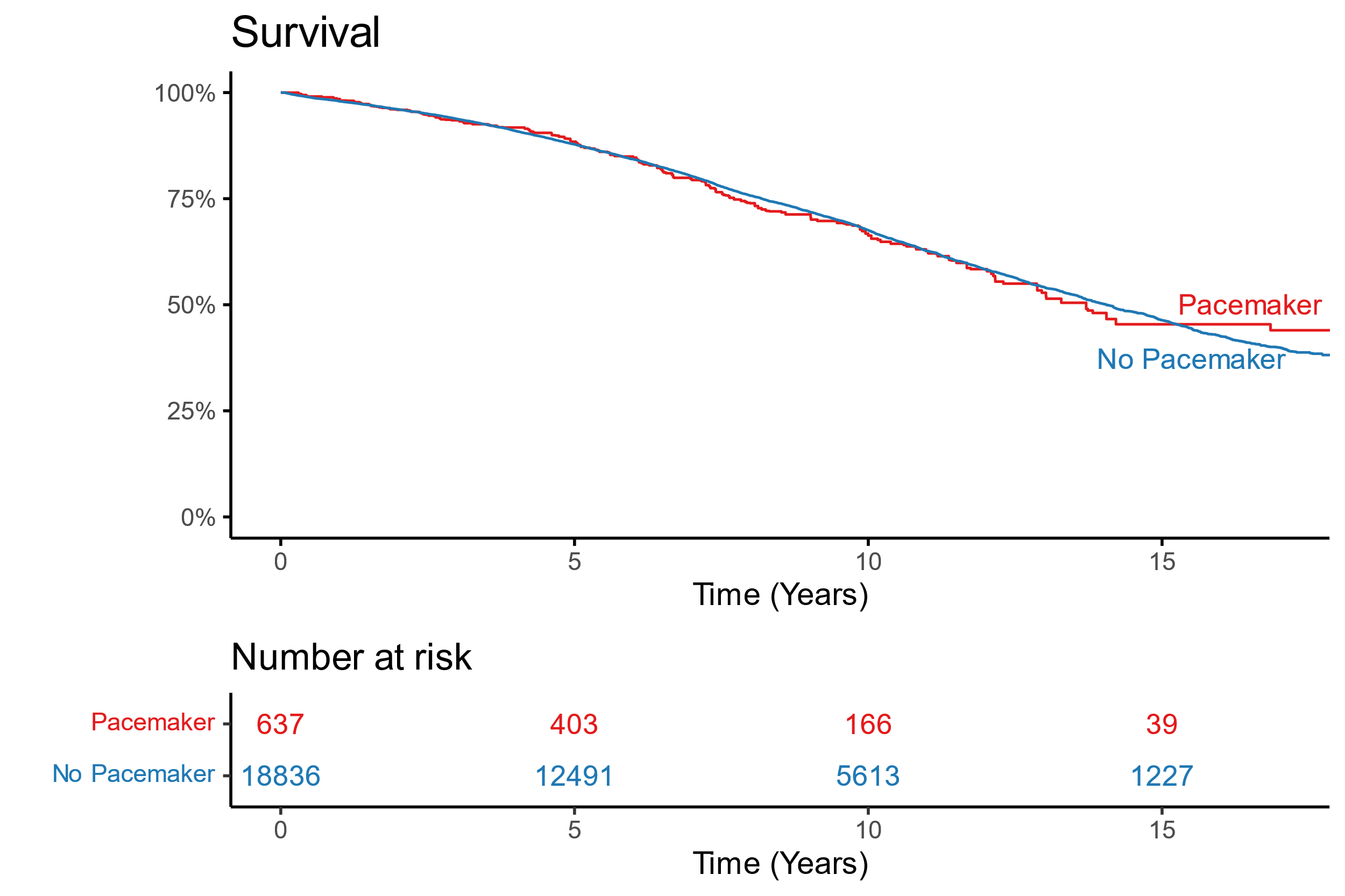


Supplemental Figure 6. Cumulative incidence of heart failure hospitalization after inverse probability of treatment weighting (estimated using flexible parametric survival models and adjusted for the competing risk of death) in patients who received permanent pacemaker implantation and those who did not receive permanent pacemaker implantation after surgical AVR in Sweden between 2001 and 2018.


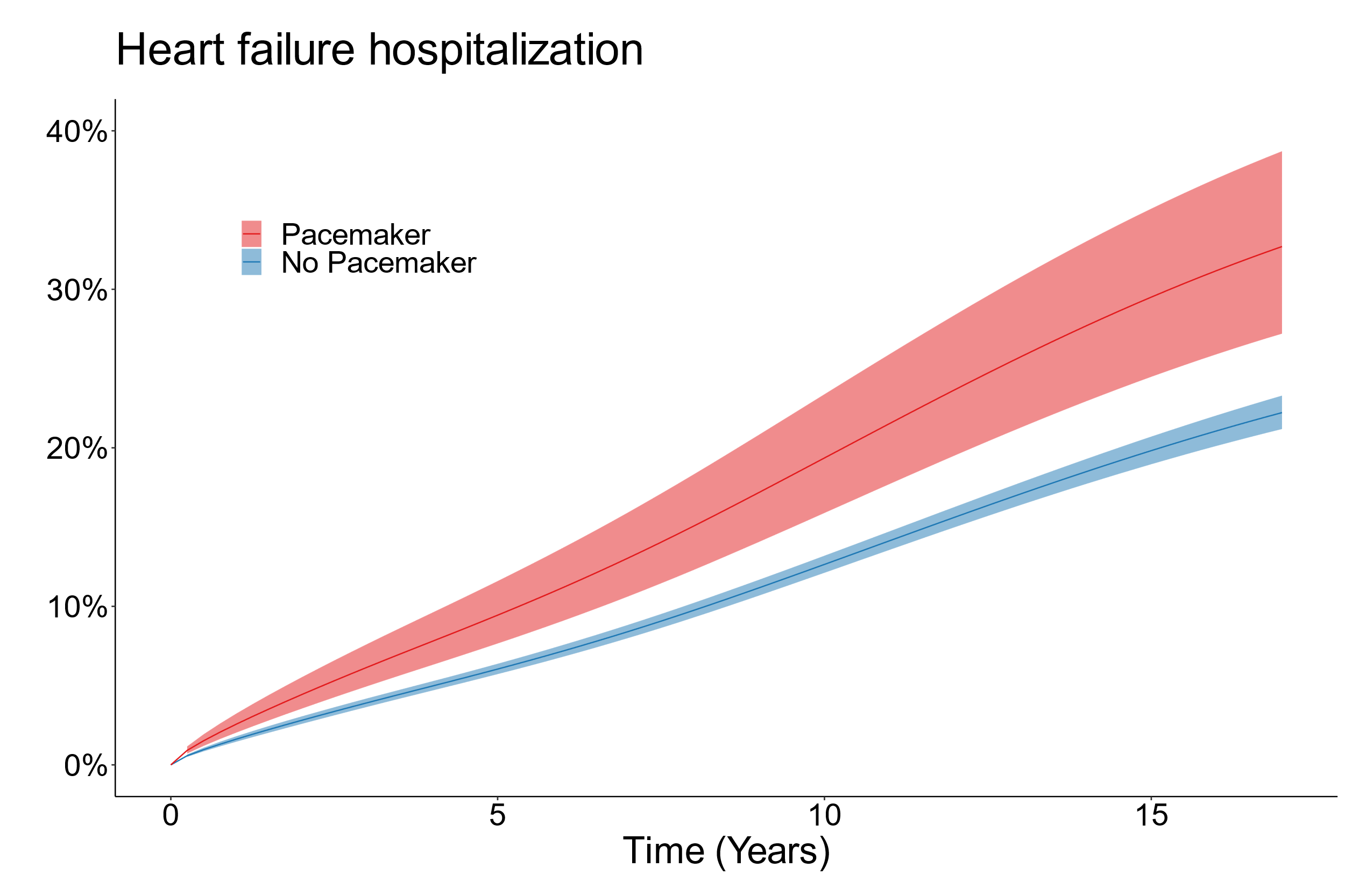


Supplemental Figure 7. Cumulative incidence of endocarditis after inverse probability of treatment weighting (estimated using flexible parametric survival models and adjusted for the competing risk of death) in patients who received permanent pacemaker implantation and those who did not receive permanent pacemaker implantation after surgical AVR in Sweden between 2001 and 2018.


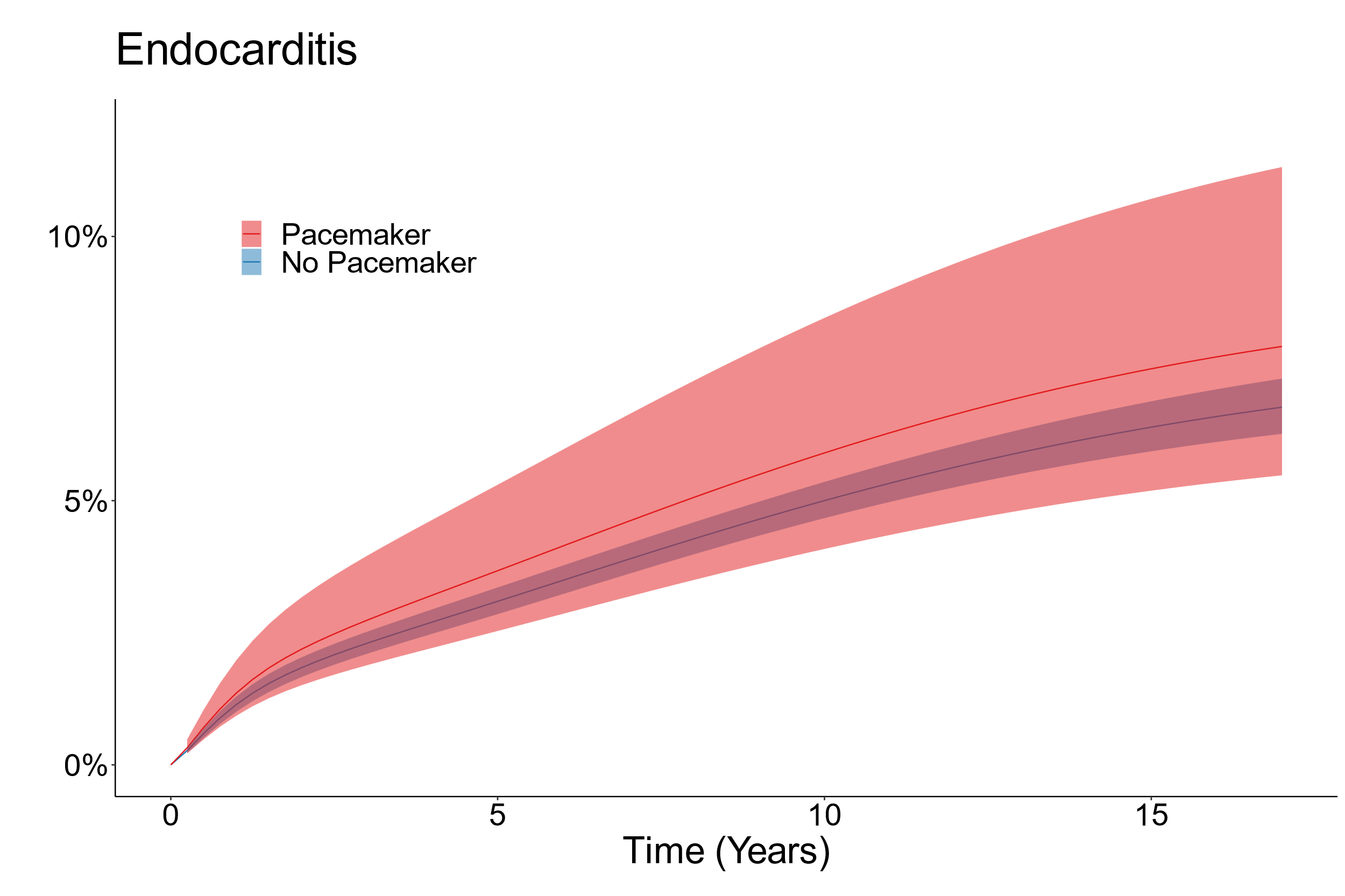


Supplemental Figure 8. Kaplan-Meier estimated crude survival in patients who received permanent pacemaker implantation and those who did not receive permanent pacemaker implantation after surgical AVR in Sweden between 2001 and 2018.


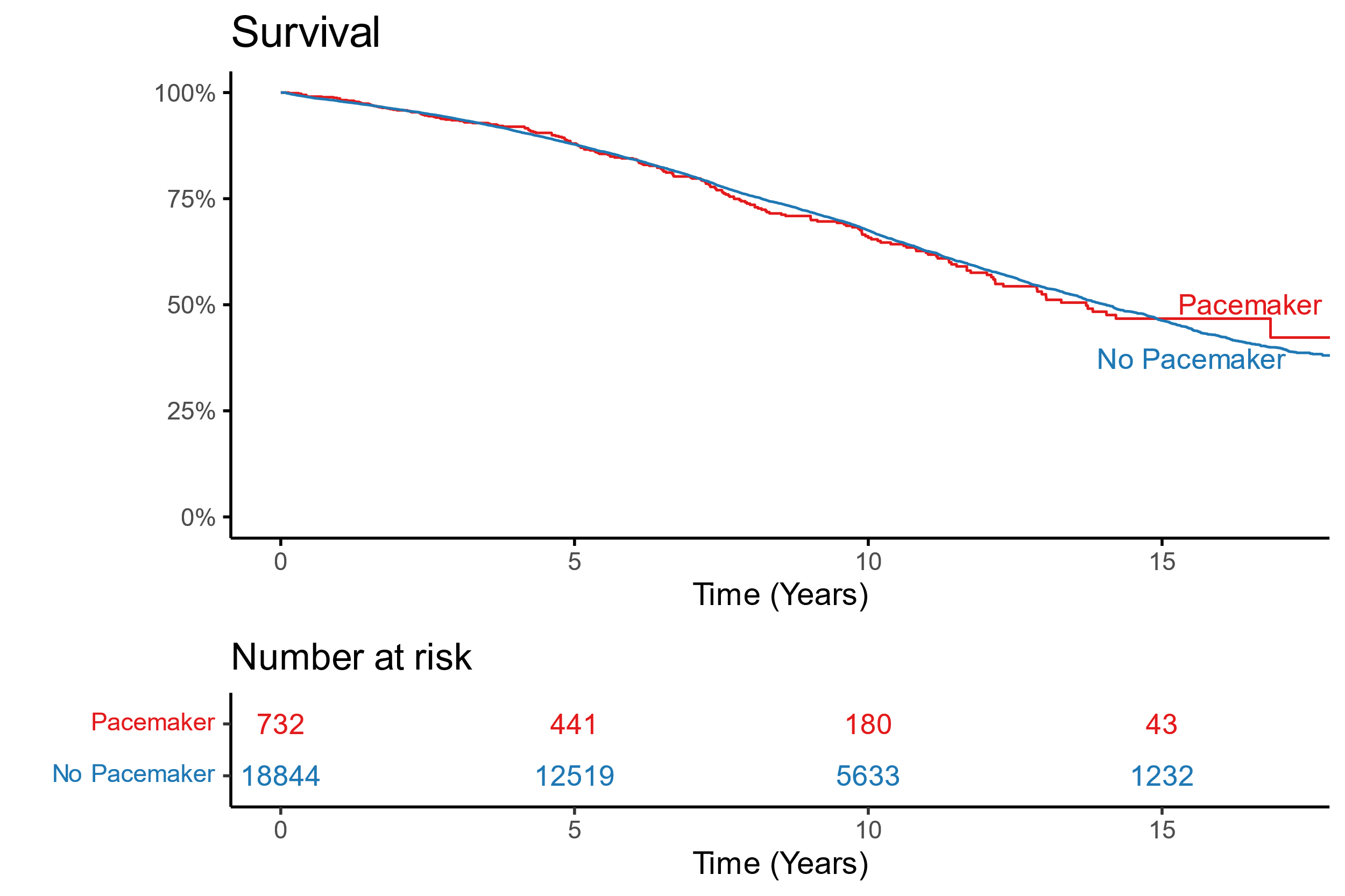


Supplemental Figure 9. Aalen-Johansen estimated crude cumulative incidence of heart failure hospitalization in patients who received or did not receive permanent pacemaker implantation after surgical AVR in Sweden between 2001 to 2018. Shaded areas represent 95% confidence intervals.


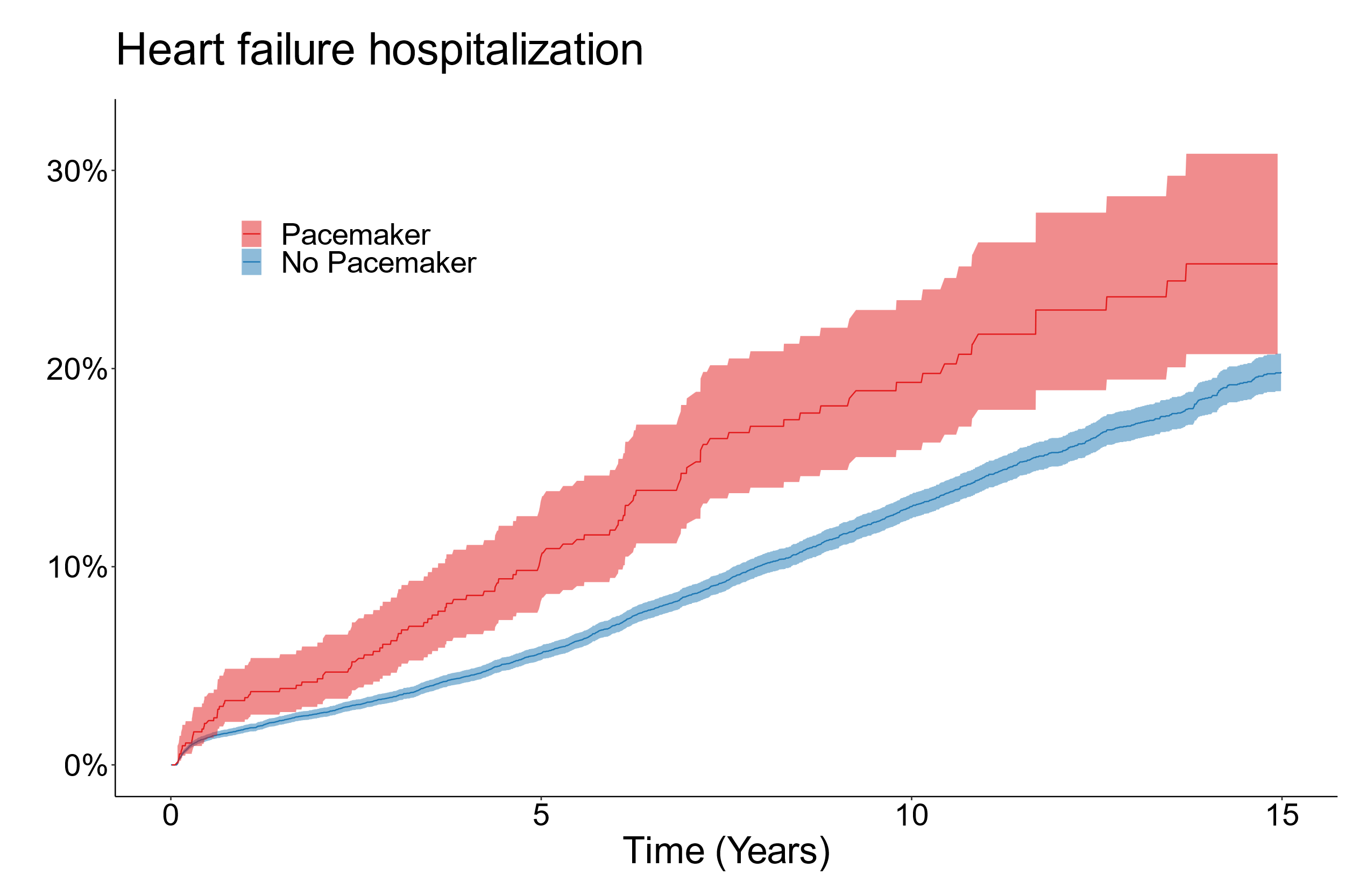


Supplemental Figure 10. Aalen-Johansen estimated crude cumulative incidence of endocarditis in patients who received or did not receive permanent pacemaker implantation after surgical AVR in Sweden between 2001 to 2018. Shaded areas represent 95% confidence intervals.

**
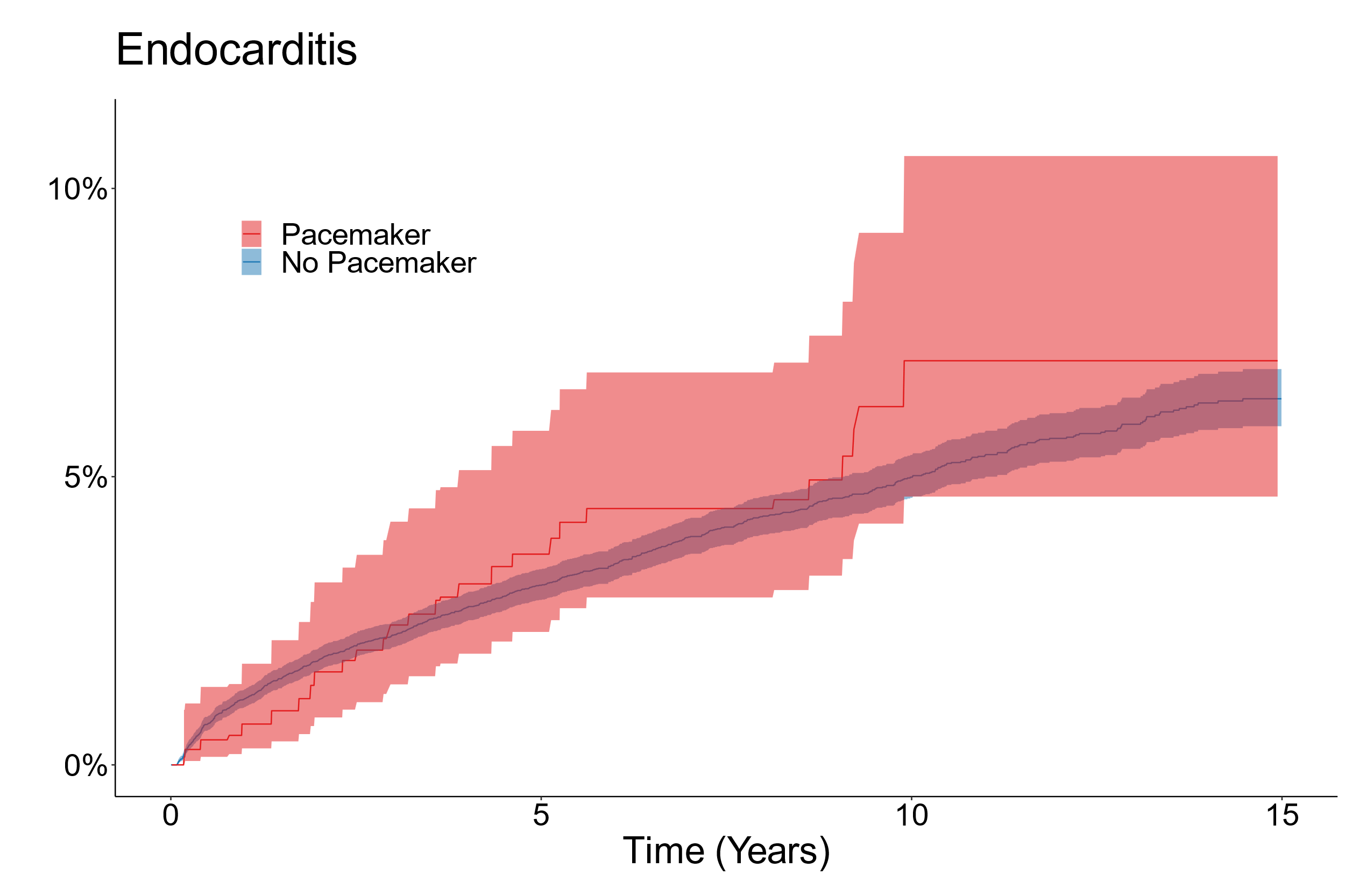
**

Supplemental Figure 11. Distribution of pacemaker implantation within 30-days following SAVR.


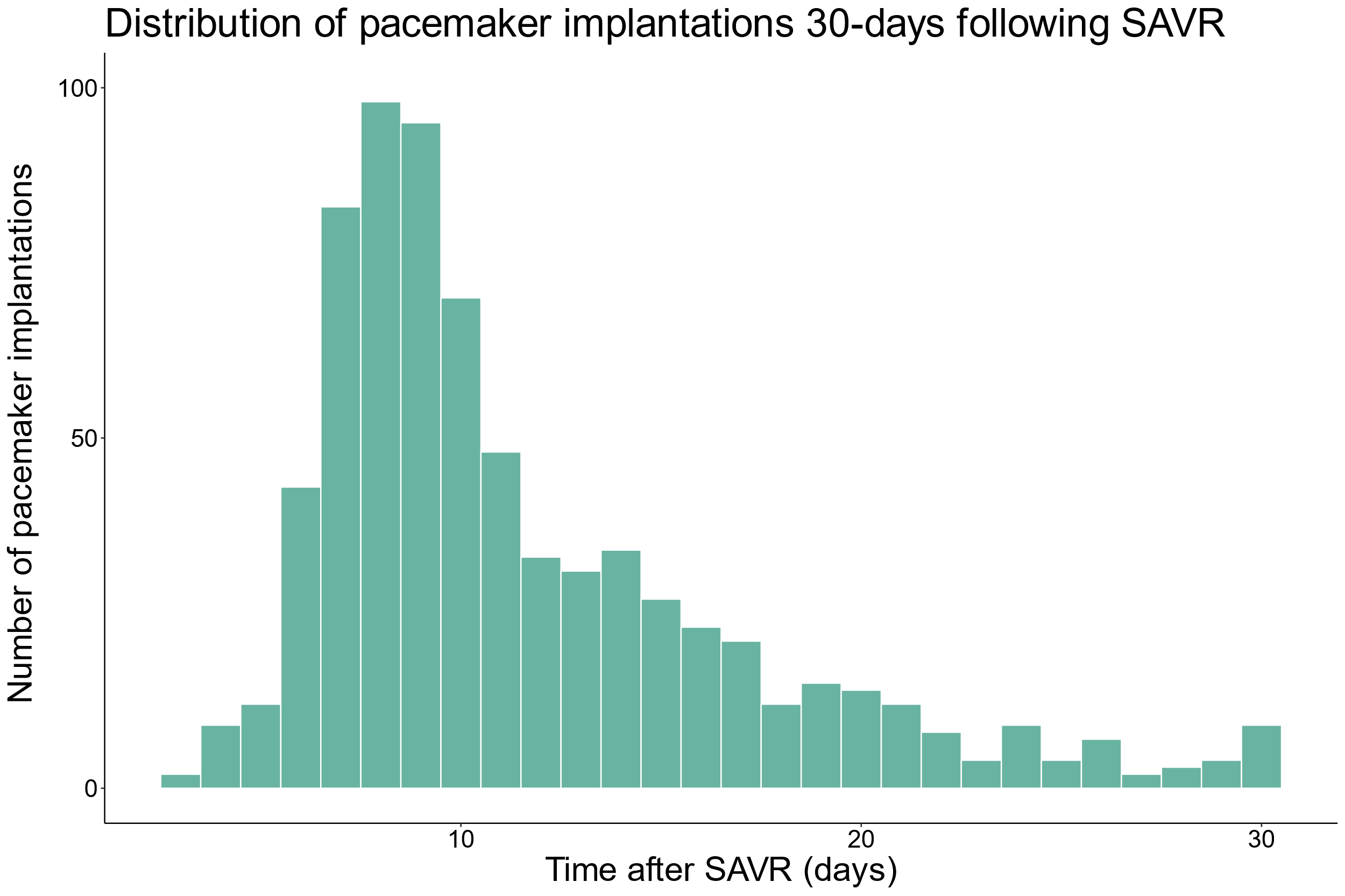

Supplement: Supplemental Data [file mmc1.docx]
